# 3,6-Dimethyl-*N* ^1^,*N* ^4^-bis­(1-phenyl­eth­yl)-1,4-dihydro-1,2,4,5-tetra­zine-1,4-dicarboxamide

**DOI:** 10.1107/S160053681200133X

**Published:** 2012-01-18

**Authors:** Guo-Wu Rao, Qi Li, Xiao-Jing Lu

**Affiliations:** a College of Pharmaceutical Science, Zhejiang University of Technology, Hangzhou, 310014, People’s Republic of China

## Abstract

In the title mol­ecule, C_22_H_26_N_6_O_2_, the central tetra­zine ring exhibits a boat conformation, and the two phenyl rings form a dihedral angle of 88.39 (6)°. In the crystal, weak N—H⋯O and C—H⋯O hydrogen bonds link mol­ecules into layers parallel to the *ab* plane.

## Related literature

For structure–activity relationships in 1,2,4,5-tetra­zine derivatives, see: Hu *et al.* (2002 ▶, 2004 ▶); Rao & Hu (2005 ▶, 2006 ▶). For standard bond lengths in organic compounds, see: Allen *et al.* (1987 ▶). For details of the synthesis, see: Hu *et al.* (2004 ▶); Skorianetz & Kovats (1970 ▶, 1971 ▶); Sun *et al.* (2003 ▶).
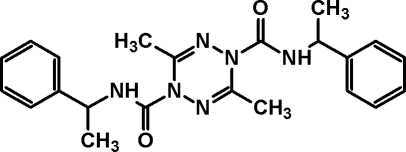


## Experimental

### 

#### Crystal data

C_22_H_26_N_6_O_2_
*M* *_r_* = 406.49
Monoclinic, 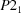

*a* = 10.4653 (15) Å
*b* = 8.0606 (12) Å
*c* = 13.711 (2) Å
β = 108.702 (1)°
*V* = 1095.5 (3) Å^3^
*Z* = 2
Mo *K*α radiation
μ = 0.08 mm^−1^
*T* = 298 K
0.37 × 0.31 × 0.26 mm

#### Data collection

Bruker SMART CCD area-detector diffractometer
Absorption correction: multi-scan (*SADABS*; Bruker, 2001 ▶) *T*_min_ = 0.970, *T*_max_ = 0.979
8343 measured reflections
4014 independent reflections
3809 reflections with *I* > 2σ(*I*)
*R*_int_ = 0.016

#### Refinement

*R*[*F* ^2^ > 2σ(*F* ^2^)] = 0.028
*wR*(*F* ^2^) = 0.072
*S* = 1.06
4014 reflections
276 parameters
1 restraint
H-atom parameters constrained
Δρ_max_ = 0.12 e Å^−3^
Δρ_min_ = −0.09 e Å^−3^

### 

Data collection: *SMART* (Bruker, 2001 ▶); cell refinement: *SAINT* (Bruker, 2001 ▶); data reduction: *SAINT*; program(s) used to solve structure: *SHELXS97* (Sheldrick, 2008 ▶); program(s) used to refine structure: *SHELXL97* (Sheldrick, 2008 ▶); molecular graphics: *ORTEP-3 for Windows* (Farrugia, 1997 ▶); software used to prepare material for publication: *WinGX* (Farrugia, 1999 ▶).

## Supplementary Material

Crystal structure: contains datablock(s) I, global. DOI: 10.1107/S160053681200133X/cv5230sup1.cif

Structure factors: contains datablock(s) I. DOI: 10.1107/S160053681200133X/cv5230Isup2.hkl

Supplementary material file. DOI: 10.1107/S160053681200133X/cv5230Isup3.cdx

Supplementary material file. DOI: 10.1107/S160053681200133X/cv5230Isup4.cml

Additional supplementary materials:  crystallographic information; 3D view; checkCIF report

## Figures and Tables

**Table 1:** Hydrogen-bond geometry (Å, °)

*D*—H⋯*A*	*D*—H	H⋯*A*	*D*⋯*A*	*D*—H⋯*A*
N6—H6⋯O1^i^	0.86	2.44	3.2497 (15)	158
N3—H3⋯O2^ii^	0.86	2.54	3.2706 (16)	144
C13—H13⋯O2^ii^	0.93	2.57	3.4701 (17)	163

Symmetry codes: (i) 
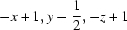
; (ii) 
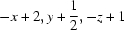
.

**Fig. 1.**
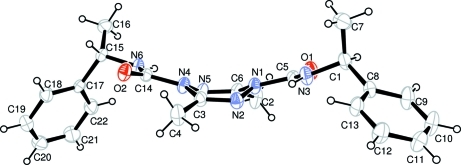
The molecular structure of (I) shown with 30% probability displacement ellipsoids.

**Fig. 2.**
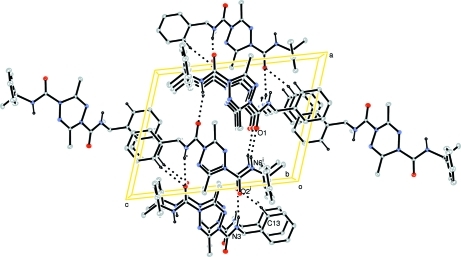
A portion of the crystal packing of (I). Hydrogen bonds are shown as dashed lines. H atoms not involved in hydrogen bonding were omitted for clarity.

## supplementary crystallographic information

### Comment

In a continuation of our studies of structure-activity relationships in 1,2,4,5-tetrazine derivatives (Hu *et al.*, 2002, 2004; Rao & Hu, 2005, 2006), we have obtained the title compound (I) as colourless crystalline solid. However, IR, NMR, and MS studies failed to prove whether the substituted groups of the nitrogen are located at the 1,4 or 1,2 positions. The structure was confirmed by single-crystal X-ray diffraction.

In (I) (Fig. 1), the N2═C3 [1.2753 (17) Å] and N5═C6 [1.2752 (18) Å] bonds correspond to typical double bonds, and the C3—N4 [1.4013 (16) Å], N4—N5 [1.4194 (15) Å], C6—N1 [1.3959 (18) Å] and N1—N2 [1.4247 (15) Å] bond lengths are typical for single bonds (Allen *et al.*, 1987). Therefore, the tetrazine ring is the 1,4-dihydro structure with the N-substituted groups at the 1,4-positions and not the 1,2-positions, the compound being 3,6-dimethyl-*N*^1^,*N*^4^-bis(1-phenylethyl)-1,2,4,5-tetrazine-1,4-dicarboxamide. Atoms N2, C3, N5 and C6 are coplanar, with the largest deviation from the plane being -0.0237 (7) Å for atom N2 and 0.0237 (7) Å for atom C6. Atoms N1 and N4 deviate from this plane by 0.3601 (21) and 0.3674 (20) Å, respectively. The dihedral angle between the N2/C3/N5/C6 plane and the N1/N2/C6 plane is 29.04 (15)°, and between the N2/C3/N5/C6 plane and the N4/N5/C3 plane is 29.72 (12)°. Therefore, the central six-member ring of the compound, the tetrazine ring, has an obvious boat conformation. The dihedral angles between the N2/C3/N5/C6 plane and the two phenyl rings are 33.24 (7) and 58.46 (6)°, respectively. The dihedral angle between the two phenyl rings is 88.39 (6)°.

In the crystal structure, weak intermolecular N—H···O and C—H···O hydrogen bonds (Table 1) link molecules into layers parallel to *ab* plane (Fig. 2).

### Experimental

The title compound was prepared according to the known procedure (Hu *et al.*, 2004; Sun *et al.*, 2003; Skorianetz *et al.*, 1970, 1971). A solution of the compound in ethanol was concentrated gradually at room temperature to afford colourless blocks (m.p. 414–416 K).

### Refinement

H atoms were included in calculated positions and refined using a riding model. H atoms were given isotropic displacement parameters equal to 1.2 (or 1.5 for methyl H atoms) times the equivalent isotropic displacement parameters of their parent atoms, and C—H distances were set to 0.96Å for methyl H atoms, 0.93 Å for phenyl H atoms, and 0.98Å for the remainder H atoms, while N—H distances were set to 0.86 Å. In the absence of any significant anomalous scatterers in the molecule, attempts to confirm the absolute structure by refinement of the Flack parameter in the presence of 1803 sets of Friedel equivalents led to an inconclusive value of 0.2 (9). Therefore, the Friedel pairs were merged before the final refinement.

### Crystal data

**Table:**

C_22_H_26_N_6_O_2_	*F*(000) = 432
*M**_r_* = 406.49	*D*_x_ = 1.232 Mg m^−^^3^
Monoclinic, *P*2_1_	Melting point = 414–416 K
Hall symbol: P 2yb	Mo *K*α radiation, λ = 0.71073 Å
*a* = 10.4653 (15) Å	Cell parameters from 5504 reflections
*b* = 8.0606 (12) Å	θ = 3.0–28.2°
*c* = 13.711 (2) Å	µ = 0.08 mm^−^^1^
β = 108.702 (1)°	*T* = 298 K
*V* = 1095.5 (3) Å^3^	Block, colourless
*Z* = 2	0.37 × 0.31 × 0.26 mm

### Data collection

**Table:**

Bruker SMART CCD area-detector diffractometer	4014 independent reflections
Radiation source: fine-focus sealed tube	3809 reflections with *I* > 2σ(*I*)
graphite	*R*_int_ = 0.016
phi and ω scans	θ_max_ = 25.5°, θ_min_ = 3.0°
Absorption correction: multi-scan (*SADABS*; Bruker, 2001)	*h* = −12→12
*T*_min_ = 0.970, *T*_max_ = 0.979	*k* = −9→9
8343 measured reflections	*l* = −16→16

### Refinement

**Table:**

Refinement on *F*^2^	Secondary atom site location: difference Fourier map
Least-squares matrix: full	Hydrogen site location: inferred from neighbouring sites
*R*[*F*^2^ > 2σ(*F*^2^)] = 0.028	H-atom parameters constrained
*wR*(*F*^2^) = 0.072	*w* = 1/[σ^2^(*F*_o_^2^) + (0.0351*P*)^2^ + 0.1082*P*] where *P* = (*F*_o_^2^ + 2*F*_c_^2^)/3
*S* = 1.06	(Δ/σ)_max_ < 0.001
4014 reflections	Δρ_max_ = 0.12 e Å^−^^3^
276 parameters	Δρ_min_ = −0.09 e Å^−^^3^
1 restraint	Extinction correction: *SHELXL97* (Sheldrick, 2008), Fc^*^=kFc[1+0.001xFc^2^λ^3^/sin(2θ)]^-1/4^
Primary atom site location: structure-invariant direct methods	Extinction coefficient: 0.079 (3)

### Special details

**Table:**

Geometry. All e.s.d.'s (except the e.s.d. in the dihedral angle between two l.s. planes) are estimated using the full covariance matrix. The cell e.s.d.'s are taken into account individually in the estimation of e.s.d.'s in distances, angles and torsion angles; correlations between e.s.d.'s in cell parameters are only used when they are defined by crystal symmetry. An approximate (isotropic) treatment of cell e.s.d.'s is used for estimating e.s.d.'s involving l.s. planes.
Refinement. Refinement of *F*^2^ against ALL reflections. The weighted *R*-factor *wR* and goodness of fit *S* are based on *F*^2^, conventional *R*-factors *R* are based on *F*, with *F* set to zero for negative *F*^2^. The threshold expression of *F*^2^ > σ(*F*^2^) is used only for calculating *R*-factors(gt) *etc*. and is not relevant to the choice of reflections for refinement. *R*-factors based on *F*^2^ are statistically about twice as large as those based on *F*, and *R*- factors based on ALL data will be even larger.

### Fractional atomic coordinates and isotropic or equivalent isotropic displacement parameters (Å^2^)

**Table:**

	*x*	*y*	*z*	*U*_iso_*/*U*_eq_	
C6	0.61215 (13)	0.43115 (19)	0.45138 (10)	0.0457 (3)	
C2	0.46716 (15)	0.3828 (3)	0.42427 (14)	0.0711 (5)	
H2A	0.4590	0.2892	0.4652	0.107*	
H2B	0.4330	0.3540	0.3526	0.107*	
H2C	0.4163	0.4742	0.4376	0.107*	
C3	0.87224 (13)	0.45508 (17)	0.46277 (10)	0.0426 (3)	
C4	1.00874 (16)	0.4220 (3)	0.45547 (13)	0.0663 (5)	
H4A	1.0746	0.4853	0.5069	0.099*	
H4B	1.0107	0.4533	0.3884	0.099*	
H4C	1.0290	0.3059	0.4664	0.099*	
C5	0.57571 (13)	0.67504 (18)	0.33409 (9)	0.0415 (3)	
C1	0.57492 (15)	0.9210 (2)	0.23003 (11)	0.0541 (4)	
H1	0.4798	0.9198	0.2263	0.065*	
C7	0.6404 (2)	1.0771 (3)	0.28855 (15)	0.0829 (6)	
H7A	0.7352	1.0766	0.2974	0.124*	
H7B	0.6274	1.0790	0.3548	0.124*	
H7C	0.5995	1.1737	0.2501	0.124*	
C8	0.58090 (13)	0.92220 (18)	0.12079 (10)	0.0443 (3)	
C9	0.46735 (16)	0.9574 (3)	0.03815 (13)	0.0680 (5)	
H9	0.3853	0.9760	0.0492	0.082*	
C10	0.47455 (17)	0.9652 (3)	−0.06069 (14)	0.0762 (6)	
H10	0.3974	0.9893	−0.1155	0.091*	
C11	0.59346 (17)	0.9381 (3)	−0.07856 (12)	0.0671 (5)	
H11	0.5977	0.9432	−0.1452	0.080*	
C12	0.70692 (17)	0.9031 (3)	0.00266 (13)	0.0667 (5)	
H12	0.7885	0.8839	−0.0089	0.080*	
C13	0.70020 (14)	0.8965 (2)	0.10129 (11)	0.0565 (4)	
H13	0.7781	0.8741	0.1558	0.068*	
C14	0.91963 (12)	0.34611 (17)	0.64199 (9)	0.0372 (3)	
C15	0.92829 (13)	0.22812 (17)	0.80845 (9)	0.0400 (3)	
H15	1.0155	0.2853	0.8314	0.048*	
C16	0.85082 (18)	0.2769 (2)	0.88085 (12)	0.0582 (4)	
H16A	0.7633	0.2259	0.8584	0.087*	
H16B	0.8408	0.3953	0.8805	0.087*	
H16C	0.8994	0.2403	0.9494	0.087*	
C17	0.95569 (12)	0.04323 (17)	0.81019 (9)	0.0381 (3)	
C18	1.08568 (15)	−0.0187 (2)	0.84585 (12)	0.0520 (4)	
H18	1.1579	0.0542	0.8700	0.062*	
C19	1.11002 (18)	−0.1875 (2)	0.84623 (14)	0.0625 (4)	
H19	1.1980	−0.2273	0.8705	0.075*	
C20	1.00479 (19)	−0.2956 (2)	0.81091 (13)	0.0660 (4)	
H20	1.0208	−0.4091	0.8111	0.079*	
C21	0.87547 (19)	−0.2360 (2)	0.77518 (16)	0.0734 (5)	
H21	0.8038	−0.3095	0.7506	0.088*	
C22	0.85099 (15)	−0.0688 (2)	0.77539 (13)	0.0582 (4)	
H22	0.7626	−0.0303	0.7518	0.070*	
N1	0.65789 (11)	0.55475 (16)	0.39939 (8)	0.0449 (3)	
N2	0.78681 (11)	0.52904 (15)	0.38757 (8)	0.0461 (3)	
N4	0.83276 (10)	0.39916 (16)	0.54557 (8)	0.0433 (3)	
N5	0.69652 (11)	0.34775 (17)	0.52228 (9)	0.0489 (3)	
N3	0.64017 (12)	0.77317 (17)	0.28704 (9)	0.0520 (3)	
H3	0.7215	0.7495	0.2901	0.062*	
N6	0.85493 (10)	0.28577 (15)	0.70385 (8)	0.0426 (3)	
H6	0.7683	0.2805	0.6819	0.051*	
O1	0.45662 (10)	0.68726 (15)	0.32671 (8)	0.0580 (3)	
O2	1.04136 (9)	0.36545 (14)	0.66573 (7)	0.0497 (3)	

### Atomic displacement parameters (Å^2^)

**Table:**

	*U*^11^	*U*^22^	*U*^33^	*U*^12^	*U*^13^	*U*^23^
C6	0.0430 (7)	0.0569 (8)	0.0349 (6)	−0.0023 (6)	0.0093 (5)	0.0085 (6)
C2	0.0442 (8)	0.0935 (14)	0.0659 (10)	−0.0094 (9)	0.0040 (7)	0.0340 (10)
C3	0.0460 (7)	0.0469 (8)	0.0368 (6)	0.0050 (6)	0.0157 (6)	0.0089 (6)
C4	0.0557 (9)	0.0945 (13)	0.0574 (9)	0.0236 (9)	0.0304 (7)	0.0280 (9)
C5	0.0440 (7)	0.0486 (8)	0.0320 (6)	0.0077 (6)	0.0122 (5)	0.0025 (6)
C1	0.0555 (8)	0.0560 (9)	0.0537 (8)	0.0184 (7)	0.0216 (7)	0.0170 (7)
C7	0.1244 (17)	0.0603 (11)	0.0657 (12)	0.0200 (12)	0.0330 (12)	0.0012 (9)
C8	0.0419 (7)	0.0415 (7)	0.0478 (7)	0.0038 (6)	0.0119 (6)	0.0127 (6)
C9	0.0425 (8)	0.0951 (14)	0.0638 (10)	0.0123 (8)	0.0133 (7)	0.0306 (10)
C10	0.0515 (9)	0.1116 (17)	0.0542 (10)	0.0021 (10)	0.0010 (7)	0.0309 (10)
C11	0.0693 (10)	0.0853 (13)	0.0443 (8)	−0.0056 (10)	0.0148 (7)	0.0120 (9)
C12	0.0543 (9)	0.0927 (13)	0.0554 (9)	0.0067 (9)	0.0209 (7)	0.0111 (9)
C13	0.0400 (7)	0.0769 (11)	0.0476 (8)	0.0076 (7)	0.0071 (6)	0.0097 (8)
C14	0.0391 (7)	0.0386 (6)	0.0332 (6)	0.0035 (5)	0.0104 (5)	0.0027 (5)
C15	0.0402 (7)	0.0460 (8)	0.0304 (6)	−0.0022 (5)	0.0068 (5)	0.0044 (5)
C16	0.0776 (11)	0.0564 (9)	0.0449 (8)	0.0074 (8)	0.0254 (7)	−0.0005 (7)
C17	0.0414 (7)	0.0457 (7)	0.0261 (6)	−0.0016 (6)	0.0093 (5)	0.0034 (5)
C18	0.0437 (8)	0.0514 (8)	0.0556 (9)	−0.0002 (6)	0.0087 (6)	0.0029 (7)
C19	0.0574 (9)	0.0585 (10)	0.0672 (10)	0.0145 (8)	0.0139 (8)	0.0049 (8)
C20	0.0843 (12)	0.0439 (9)	0.0656 (11)	0.0055 (8)	0.0182 (9)	−0.0025 (8)
C21	0.0668 (11)	0.0516 (10)	0.0899 (14)	−0.0146 (9)	0.0084 (9)	−0.0079 (9)
C22	0.0436 (8)	0.0546 (10)	0.0683 (9)	−0.0037 (7)	0.0064 (7)	−0.0005 (8)
N1	0.0406 (6)	0.0557 (7)	0.0404 (6)	0.0081 (5)	0.0156 (5)	0.0130 (5)
N2	0.0447 (6)	0.0567 (7)	0.0412 (6)	0.0093 (5)	0.0198 (5)	0.0124 (5)
N4	0.0366 (5)	0.0573 (7)	0.0352 (5)	−0.0019 (5)	0.0103 (4)	0.0103 (5)
N5	0.0384 (6)	0.0635 (7)	0.0407 (6)	−0.0066 (5)	0.0069 (5)	0.0153 (6)
N3	0.0487 (7)	0.0588 (8)	0.0535 (7)	0.0166 (6)	0.0232 (6)	0.0212 (6)
N6	0.0352 (6)	0.0533 (7)	0.0366 (6)	0.0004 (5)	0.0078 (4)	0.0116 (5)
O1	0.0446 (6)	0.0702 (7)	0.0606 (7)	0.0120 (5)	0.0189 (5)	0.0167 (6)
O2	0.0371 (5)	0.0695 (7)	0.0409 (5)	0.0005 (5)	0.0104 (4)	0.0072 (5)

### Geometric parameters (Å, °)

**Table:**

C6—N5	1.2752 (18)	C11—H11	0.9300
C6—N1	1.3959 (18)	C12—C13	1.377 (2)
C6—C2	1.4935 (19)	C12—H12	0.9300
C2—H2A	0.9600	C13—H13	0.9300
C2—H2B	0.9600	C14—O2	1.2195 (15)
C2—H2C	0.9600	C14—N6	1.3351 (17)
C3—N2	1.2753 (17)	C14—N4	1.4094 (16)
C3—N4	1.4013 (16)	C15—N6	1.4684 (16)
C3—C4	1.4879 (19)	C15—C17	1.516 (2)
C4—H4A	0.9600	C15—C16	1.5210 (19)
C4—H4B	0.9600	C15—H15	0.9800
C4—H4C	0.9600	C16—H16A	0.9600
C5—O1	1.2217 (16)	C16—H16B	0.9600
C5—N3	1.3326 (18)	C16—H16C	0.9600
C5—N1	1.4098 (17)	C17—C22	1.382 (2)
C1—N3	1.4678 (19)	C17—C18	1.383 (2)
C1—C8	1.519 (2)	C18—C19	1.384 (2)
C1—C7	1.531 (3)	C18—H18	0.9300
C1—H1	0.9800	C19—C20	1.366 (3)
C7—H7A	0.9600	C19—H19	0.9300
C7—H7B	0.9600	C20—C21	1.370 (3)
C7—H7C	0.9600	C20—H20	0.9300
C8—C13	1.3738 (19)	C21—C22	1.372 (3)
C8—C9	1.383 (2)	C21—H21	0.9300
C9—C10	1.383 (2)	C22—H22	0.9300
C9—H9	0.9300	N1—N2	1.4247 (15)
C10—C11	1.361 (2)	N4—N5	1.4194 (15)
C10—H10	0.9300	N3—H3	0.8600
C11—C12	1.372 (2)	N6—H6	0.8600
			
N5—C6—N1	119.91 (12)	C8—C13—H13	119.3
N5—C6—C2	116.88 (13)	C12—C13—H13	119.3
N1—C6—C2	123.12 (12)	O2—C14—N6	125.18 (11)
C6—C2—H2A	109.5	O2—C14—N4	121.06 (11)
C6—C2—H2B	109.5	N6—C14—N4	113.60 (11)
H2A—C2—H2B	109.5	N6—C15—C17	111.31 (11)
C6—C2—H2C	109.5	N6—C15—C16	109.54 (12)
H2A—C2—H2C	109.5	C17—C15—C16	112.43 (12)
H2B—C2—H2C	109.5	N6—C15—H15	107.8
N2—C3—N4	119.54 (12)	C17—C15—H15	107.8
N2—C3—C4	117.96 (12)	C16—C15—H15	107.8
N4—C3—C4	122.39 (12)	C15—C16—H16A	109.5
C3—C4—H4A	109.5	C15—C16—H16B	109.5
C3—C4—H4B	109.5	H16A—C16—H16B	109.5
H4A—C4—H4B	109.5	C15—C16—H16C	109.5
C3—C4—H4C	109.5	H16A—C16—H16C	109.5
H4A—C4—H4C	109.5	H16B—C16—H16C	109.5
H4B—C4—H4C	109.5	C22—C17—C18	117.87 (14)
O1—C5—N3	124.99 (12)	C22—C17—C15	120.80 (12)
O1—C5—N1	120.43 (12)	C18—C17—C15	121.32 (12)
N3—C5—N1	114.53 (11)	C17—C18—C19	121.00 (15)
N3—C1—C8	111.78 (12)	C17—C18—H18	119.5
N3—C1—C7	109.55 (13)	C19—C18—H18	119.5
C8—C1—C7	111.00 (14)	C20—C19—C18	119.98 (16)
N3—C1—H1	108.1	C20—C19—H19	120.0
C8—C1—H1	108.1	C18—C19—H19	120.0
C7—C1—H1	108.1	C19—C20—C21	119.65 (16)
C1—C7—H7A	109.5	C19—C20—H20	120.2
C1—C7—H7B	109.5	C21—C20—H20	120.2
H7A—C7—H7B	109.5	C20—C21—C22	120.49 (16)
C1—C7—H7C	109.5	C20—C21—H21	119.8
H7A—C7—H7C	109.5	C22—C21—H21	119.8
H7B—C7—H7C	109.5	C21—C22—C17	121.00 (15)
C13—C8—C9	117.85 (14)	C21—C22—H22	119.5
C13—C8—C1	121.22 (12)	C17—C22—H22	119.5
C9—C8—C1	120.85 (13)	C6—N1—C5	125.16 (11)
C10—C9—C8	120.65 (15)	C6—N1—N2	116.52 (11)
C10—C9—H9	119.7	C5—N1—N2	115.78 (10)
C8—C9—H9	119.7	C3—N2—N1	114.83 (10)
C11—C10—C9	120.62 (15)	C3—N4—C14	126.05 (11)
C11—C10—H10	119.7	C3—N4—N5	116.58 (10)
C9—C10—H10	119.7	C14—N4—N5	114.94 (10)
C10—C11—C12	119.37 (16)	C6—N5—N4	114.68 (11)
C10—C11—H11	120.3	C5—N3—C1	121.22 (12)
C12—C11—H11	120.3	C5—N3—H3	119.4
C11—C12—C13	120.10 (15)	C1—N3—H3	119.4
C11—C12—H12	119.9	C14—N6—C15	121.52 (11)
C13—C12—H12	119.9	C14—N6—H6	119.2
C8—C13—C12	121.39 (14)	C15—N6—H6	119.2
			
N3—C1—C8—C13	−50.2 (2)	O1—C5—N1—C6	7.0 (2)
C7—C1—C8—C13	72.40 (19)	N3—C5—N1—C6	−175.44 (14)
N3—C1—C8—C9	132.96 (16)	O1—C5—N1—N2	168.28 (13)
C7—C1—C8—C9	−104.41 (19)	N3—C5—N1—N2	−14.20 (18)
C13—C8—C9—C10	0.4 (3)	N4—C3—N2—N1	3.17 (19)
C1—C8—C9—C10	177.27 (18)	C4—C3—N2—N1	179.28 (15)
C8—C9—C10—C11	0.2 (3)	C6—N1—N2—C3	−34.68 (18)
C9—C10—C11—C12	−0.2 (3)	C5—N1—N2—C3	162.40 (13)
C10—C11—C12—C13	−0.3 (3)	N2—C3—N4—C14	−166.55 (13)
C9—C8—C13—C12	−0.8 (3)	C4—C3—N4—C14	17.5 (2)
C1—C8—C13—C12	−177.74 (17)	N2—C3—N4—N5	32.1 (2)
C11—C12—C13—C8	0.8 (3)	C4—C3—N4—N5	−143.84 (16)
N6—C15—C17—C22	−57.93 (17)	O2—C14—N4—C3	9.7 (2)
C16—C15—C17—C22	65.39 (16)	N6—C14—N4—C3	−174.56 (14)
N6—C15—C17—C18	121.65 (14)	O2—C14—N4—N5	171.31 (13)
C16—C15—C17—C18	−115.02 (15)	N6—C14—N4—N5	−12.93 (17)
C22—C17—C18—C19	0.3 (2)	N1—C6—N5—N4	3.8 (2)
C15—C17—C18—C19	−179.33 (15)	C2—C6—N5—N4	−179.63 (15)
C17—C18—C19—C20	0.1 (3)	C3—N4—N5—C6	−35.42 (19)
C18—C19—C20—C21	0.0 (3)	C14—N4—N5—C6	161.14 (13)
C19—C20—C21—C22	−0.5 (3)	O1—C5—N3—C1	7.4 (2)
C20—C21—C22—C17	0.9 (3)	N1—C5—N3—C1	−169.98 (13)
C18—C17—C22—C21	−0.7 (2)	C8—C1—N3—C5	−126.84 (15)
C15—C17—C22—C21	178.87 (16)	C7—C1—N3—C5	109.70 (17)
N5—C6—N1—C5	−167.49 (14)	O2—C14—N6—C15	−2.9 (2)
C2—C6—N1—C5	16.2 (2)	N4—C14—N6—C15	−178.49 (12)
N5—C6—N1—N2	31.4 (2)	C17—C15—N6—C14	−93.33 (15)
C2—C6—N1—N2	−144.94 (16)	C16—C15—N6—C14	141.71 (14)

### Hydrogen-bond geometry (Å, °)

**Table:**

*D*—H···*A*	*D*—H	H···*A*	*D*···*A*	*D*—H···*A*
N6—H6···O1^i^	0.86	2.44	3.2497 (15)	158
N3—H3···O2^ii^	0.86	2.54	3.2706 (16)	144
C13—H13···O2^ii^	0.93	2.57	3.4701 (17)	163

Symmetry codes: (i) −*x*+1, *y*−1/2, −*z*+1; (ii) −*x*+2, *y*+1/2, −*z*+1.
